# Changes in Sagittal Balance of Motion at the Thumb Metacarpophalangeal Joint After Sequential Trapeziectomy, Suspensionplasty, and Extensor Pollicis Brevis Tenodesis: A Cadaveric Study

**DOI:** 10.1177/15589447251339505

**Published:** 2025-06-12

**Authors:** Javier de Torres-Urrea, Olga Roda, Alejandro Molina-Molina, Victor M Soto, Clarisa Simón-Pérez, Indalecio Sánchez-Montesinos, Pedro Hernández-Cortés

**Affiliations:** 1 Hospital Mediterraneo, Almería, Spain; 2 University of Granada, Spain; 3 Sport and Health University Research Institute, Granada, Spain; 4 University Hospital of Valladolid, Spain; 5 Instituto de Investigación Biosanitaria (IBS), Granada, Spain; 6 San Cecilio University Hospital, Granada, Spain

**Keywords:** thumb, metacarpophalangeal joint, thumb deformity, palmar plate, tenodesis, trapezium bone, osteoarthritis

## Abstract

**Background:**

We hypothesized that hyperextension metacarpophalangeal (MP) thumb deformity may be caused by trapeziectomy alone due to telescoping of the first metacarpal without trapezial support.

**Objective:**

To explore the relationship of trapeziectomy with hyperextension thumb MP joint deformity and contribute novel data on the underlying pathomechanics.

**Methods:**

This basic anatomical research study examined thumb MP joint kinematics at 6 surgical stages in 10 cadaveric specimens: stage 1, baseline; 2, volar plate of the thumb MP joint division; 3, trapeziectomy; 4, Weilby’s ligament reconstruction and tendon interposition (LRTI); 5, extensor pollicis brevis (EPB) tenotomy; and 6, MP volar plate reconstruction by tenodesis with EPB. Six infrared cameras were used in a motion capture system to determine three-dimensional angles of the first MP joint during 10 cycles of thumb flexion-extension, measuring angles in maximum flexion and extension and the complete flexion-extension arc.

**Results:**

In comparison to baseline, the angle in extension and MP-ROM were significantly increased after stages 3 (trapezium extraction), 4 (LRTI), and 5 (EPB tenotomy). In comparison to values after stages 3, 4, and 5, the hyperextension was significantly corrected after stage 6 (tenodesis).

**Conclusions:**

In a cadaveric model of trapeziectomy, secondary thumb column shortening favors an MP joint hyperextension deformity that is not corrected by LRTI or EPB tenotomy and requires a stabilization procedure.

## Introduction

Hyperextension deformity of the metacarpophalangeal (MP) joint of the thumb can be innate, posttraumatic, or neurological, and it is frequently associated with trapeziometacarpal (TM) osteoarthritis. This deformity often contributes to thumb pain and loss of function^1^ and has been described as a poor prognostic factor for trapeziectomy.^2^ Nevertheless, the need for MP joint treatment in patients with TM osteoarthritis and the ideal approach remain controversial. Some authors recommend the simultaneous treatment of TM and MP joints in patients with TM osteoarthritis.^3-5^ However, others found no statistically significant differences in postsurgical functional outcomes between patients without preoperative MP hyperextension and patients with untreated hyperextension, especially when the deformity is less than 30°.^6^

Metacarpophalangeal joint hyperextension has been considered as a compensatory mechanism in advanced TM osteoarthritis to improve the hand span, but it also weakens the pinch force.^7,8^ However, several authors have proposed that sagittal MP joint instability is directly due to thumb shortening and TM subluxation.^3,9,10^ We hypothesized that MP joint hyperextension may also occur after trapeziectomy due to proximal migration of the first metacarpal without trapezial support.

Various questions need to be addressed: whether trapeziectomy and consequent thumb shortening alone foster MP joint deformity, whether ligament reconstruction and tendon interposition (LRTI) is effective to prevent gradual “zigzag” collapse, and whether simultaneous MP flexion tenodesis and trapeziectomy affect the balance of motion at the thumb. The purpose of our study was to determine the relationship between trapeziectomy and MP joint hyperextension and to explore the modifying effects of: (1) extensor pollicis brevis (EPB) tenotomy; (2) LTRI; and (3) tenodesis with EPB.

## Material and Methods

### Design

This basic anatomical research study investigated thumb MP joint kinematics in cadaveric specimens at six surgical stages. It followed the ethical principles of biomedical research and was approved by the Ethics Committee for Human Research of the University of Granada (Spain) (Cod: 3680/CEIH/2023).

### Sample

The study included 10 forearm and hand specimens from 7 fresh-frozen cadavers (3 males and 4 females) aged between 63 and 82 years at death, who had all given informed consent to the use of their bodies for scientific purposes.

Selected specimens (5 right and 5 left forearm and hand specimens) had no documented wrist or hand injury or surgery, nor preexisting deformities or instabilities at the MP joint, and X-ray examination ruled out signs of thumb carpometacarpal or MP osteoarthritis. Specimens were thawed at room temperature and dissected in the Human Anatomy Department of the School of Medicine of the University of Granada (Spain) between March and May 2023.

### Experimental Setup

**Figure 1.**
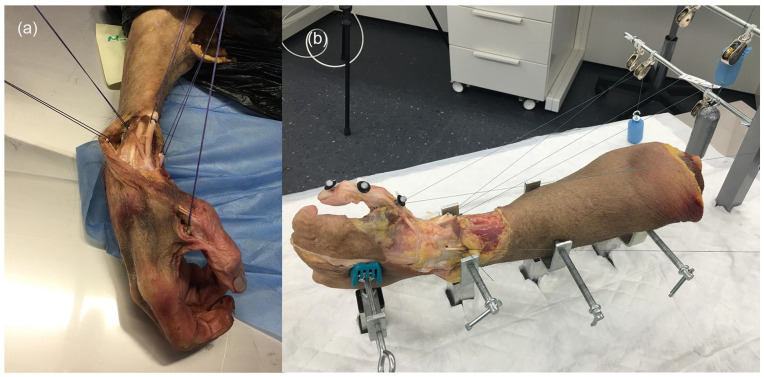
Placement of the specimen on the experimental table. *Note.* (a) Identification and labeling with sutures of the tendons that mobilize the thumb. (b) Placement of the specimen on the experimental table. The loading of the tendons is done with weights on pulleys. Three reflectors are implanted in the column of the thumb for the identification of movement by the cameras and calculation of the angle of the metacarpophalangeal joint.

The experimental model followed that used by Imaeda et al^11^ and Koff et al.^12^ Tendons of the extensor pollicis longus (EPL), flexor pollicis longus (FPL), abductor pollicis longus (APL), and EPB were isolated in forearm proximal to their natural pulley in the extensor retinaculum. The adductor pollicis (AP) and abductor pollicis brevis (APB) were detached from their origins. Tendons of dissected muscle groups were then transfixed with the corresponding sutures for traction, reproducing the physiological direction of the vector of each muscle (Figure 1a). Each specimen was fixed to a metallic device (Figure 1b). A constant load of 50 g was applied to each extrinsic and intrinsic tendon to stabilize the TM joint, recreating thumb column flexion-extension movements. The simultaneous loading of EPL, EPB, and APL produced a net extension moment, while the simultaneous loading of APL, AP, and FPL produced a flexion moment.

**Figure 2.**
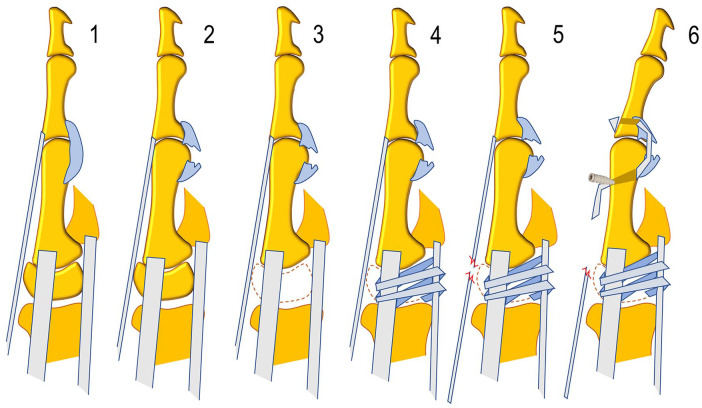
Experimental stages: 1. Unaltered specimen, 2. division of the volar plate, 3. trapeziectomy, 4. ligamentous reconstruction and tendon interposition by Weilby technique, 5. tenotomy of the extensor pollicis brevis, 6. tenodesis with extensor pollicis brevis.

Kinematic tests of the thumb MP joint were performed at baseline with the joint intact (stage 1) and then, after volar plate division, to experimentally simulate the palmar plate attenuation required to develop hyperextension deformity (stage 2); trapeziectomy (stage 3); Weilby’s LRTI (stage 4)^13,14^; EPB tenotomy, to rule out EPB as a contributor to the deformity (stage 5); and MP volar tenodesis^15^ at 20° of flexion, measured by analog goniometer (stage 6) (Figure 2).

### Testing Methods

**Figure 3.**
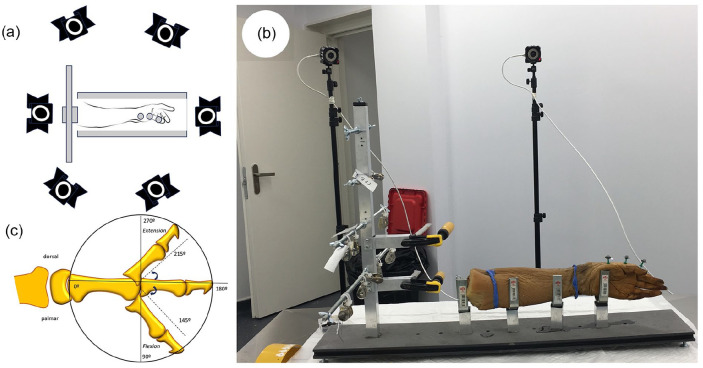
Metacarpophalangeal mobility measurement system. *Note.* (a) Diagram of the distribution of the cameras around the experimental table. (b) Photograph of the experiment table and arrangement of the 3 reflectors on the thumb column. (c) Way of measuring the metacarpophalangeal angle. Hyperextension is in the arc from 180° to 360°, and flexion between 180° and 0°.

Angles of the first MP joint were measured during 10 cycles of thumb flexion-extension while the thumb had a load applied with a Prime 41 six-infrared-camera motion capture system (OptiTrack, NaturalPoint, Corvallis, Oregon) at 180 Hz and a resolution of 2048 × 2048 pixels, using Motive software v.2.3. (OptiTrack, NaturalPoint, Corvallis, Oregon; Figure 3a) for kinetic data collection. The angle of the first MP joint was derived from three passive reflective markers (Figure 3b) on the dorsum of the metacarpal and proximal and distal phalanges of the thumb. The absence of wires and small size of markers (spheres with diameter of 4 mm) allowed fingers to be moved without limitations. Joint angles were computed from the kinematic data using Kwon 3D software (Visol., Inc., Seoul, Korea). A fourth-order Butterworth low-pass filter was applied, setting the cut-off frequency at 12 Hz. The deformity was analyzed in the sagittal plane alone. Study variables were MP joint angle in maximum flexion and in maximum extension and the complete flexion-extension arc (MP-ROM), considering the mean value of 10 angle measurements at maximum flexion and maximum extension.

### Statistical Analysis

Data for study variables were entered in a mixed linear model with two factors: hand (random effects) and stage (fixed effects). Global analysis was followed by paired comparisons for each stage, calculating the effect size with standard error and confidence interval. Given the exploratory nature of this study, pairwise comparisons were not penalized. STATA 14-0 (Stata Press, TX 77845) was used for statistical analysis, considering *P* < .05 to be significant.

## Results

**Figure 4.**
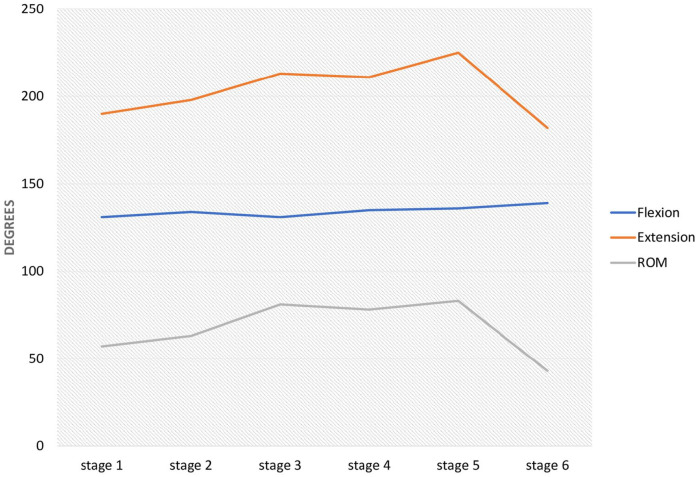
Mean angle of the metacarpophalangeal joint with thumb flexion, extension, and range of overall mobility by experimental stages. *Note.* ROM = range of flexion-extension motion.

Results are summarized in Tables 1 and 2 and Figure 4.

**Table 1.** Arc of Movement of Metacarpophalangeal Joint of the Thumb Through the Experimental Stages.

Variable	Mean	SD	Min	Max
Flexion stage 1	130.8	13.03	118	149
Flexion stage 2	134.3	22.36	107	174
Flexion stage 3	131.4	21.49	100	171
Flexion stage 4	135.5	27.17	105	167
Flexion stage 5	136.2	16.89	110	154
Flexion stage 6	139.2	13.68	116	150
Ext. stage 1	189.9	24.69	158	215
Ext. stage 2	197.7	23.15	160	215
Ext. stage 3	213.1	17.37	190	237
Ext. stage 4	211.0	11.14	201	225
Ext. stage 5	225.3	18.13	202	254
Ext. stage 6	182.2	17.43	159	197
ROM stage 1	57.3	21.54	33	92
ROM stage 2	63.1	23.08	33	94
ROM stage 3	81.2	20.09	63	110
ROM stage 4	77.8	25.40	53	103
ROM stage 5	82.6	22.91	48	106
ROM stage 6	43.0	23.53	21	78

*Note.* Mean range of motion of thumb metacarpophalangeal joint (in degrees) by surgical stage. SD = standard deviation; Min = minimum value; Max = maximum value; Ext. = extension; ROM = range of flexion-extension motion (see Figure 2). Stage 1: unaltered specimen; stage 2: division of volar plate; stage 3: trapeziectomy; stage 4: Weilby ligament reconstruction and tendon interposition; stage 5: EPB tenotomy; stage 6: EPB tenodesis.

**Table 2.** Statistical Comparison of the Arc of Movement of Metacarpophalangeal Joint of the Thumb Through the Experimental Stages.

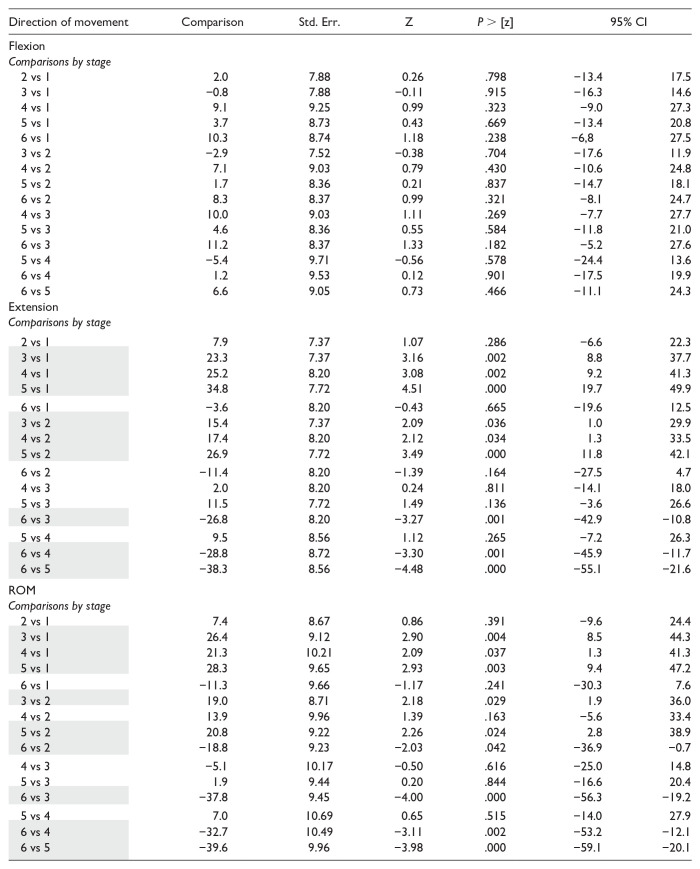						

*Note.* Statistical comparison of ranges of motion by surgical stage. Std. Err = standard error; Z = Z statistic; 95% CI = 95% confidence interval; ROM = range of flexion-extension motion. Stage 1: unaltered specimen; stage 2: division of the volar plate; stage 3: trapeziectomy; stage 4: Weilby ligament reconstruction and tendon interposition; stage 5: EPB division; stage 6: EPB tenotomy. Shaded cell = statistically significant comparison.

At baseline, the mean thumb MP angle was 130.80° ± 13.03° in flexion and 189.90° ± 24.69° in extension; it increased to 197.70° ± 23.15° in extension after volar plate division, and further to 213.10° ± 17.37° after trapeziectomy. The mean maximum extension changed a little after LRTI (211.0° ± 11.14°) and EPB tenotomy (225.3° ± 18.13°) but was reduced to 182.2° ± 17.43° by EPB tenodesis.

Maximum flexion results did not significantly vary among the 6 stages (Table 2), but there were differences in maximum extension and MP-ROM values. Volar plate division (stage 2) did not significantly change the maximum extension of the MP joint or the MP-ROM. However, these values were significantly increased after trapeziectomy (stage 3), and this difference persisted after LRTI by Weilby technique (stage 4) and EPB tenotomy (stage 5), with no significant difference among stages 3, 4, and 5. The hyperextension was significantly corrected by EPB tenodesis in comparison to these three stages, achieving extension and MP-ROM values that did not significantly differ from those at baseline (Table 2).

## Discussion

Metacarpophalangeal joint instability is frequently observed in the setting of thumb TM joint arthrosis,^3,16^ but the underlying pathomechanics has not been fully elucidated. It has yet to be established whether this deformity is corrected by TM joint treatment or whether its postoperative persistence impacts rhizarthrosis surgery outcomes,^2,3,16^ supporting the simultaneous treatment of MP and TM joints.^4,17,18^

In this kinematic study of the thumb MP joint in a cadaverous model, trapeziectomy significantly increased MP extension in comparison to a complete and aligned thumb column, and the deformity was not corrected by the Weilby LRT technique^13,14^or by EPB tenotomy. It was only corrected by intervention on the volar plate of the MP joint (EPB tenodesis).^15^

In our cadaveric biomechanical model, thumb MCP joint hyperextension was measured under loads previously applied by multiple authors,^11,12,19^ while higher loads have been used in similar studies on pinch force.^18^ The aforementioned authors used an electromagnetic tracking device (3Space Tracker System; Polhemus, Colchester, Vermont) to measure the range of motion of the thumb, whereas the present investigation employed an optical tracking system (OptiTrack, NaturalPoint, Corvallis, Oregon) described as a reliable and valid tool for this purpose.^20,21^

Hyperextension MP joint deformity has been described as a poor prognostic factor for trapeziectomy with LRTI^22^; however, Poulter and Davis,^17^ Brogan et al,^6^ and Pogliacomi et al^23^ found no association between preoperative MP joint hyperextension <30° and a poor outcome, even when untreated. Pain outcomes cannot be evaluated in a cadaveric model; however, one study found that the key pinch force was reduced by 53 N (4.4%) for every 10° increase in thumb MP joint hyperextension,^19^ and the loss of grip and pinch force has an evident negative impact on the quality of life of patients.^22^ Randomized trials are needed to determine the influence of persistent MP joint instability on the clinical outcomes of trapeziectomy and LRTI.

Little research has been published on the effect of suspensionplasty on preoperative MP joint hyperextension. Komura et al^22^ reported that LRTI with flexor carpi radialis (FCR) hemitendon through the bone tunnel improves hyperextension deformity >30°, but their retrospective study included only 28 cases of trapeziectomy with LRTI, and just 9 of these had a deformity >30°. Their results conflict with findings of an increase in MP hyperextension during the follow-up of patients undergoing trapeziectomy and LRTI with FCR tendon,^24^ and with the present observation that hyperextension and range of motion were elevated after trapeziectomy versus baseline and did not significantly differ from the values observed after Weilby suspensionplasty. It may therefore be concluded that suspensionplasty is not effective to prevent gradual “zigzag” collapse of the thumb.

Conversely, the present results suggest that trapeziectomy and the consequent thumb shortening alone play a major role in the development of MP joint deformity. Some classic studies directly attributed MP joint instability to thumb shortening and TM subluxation.^3,9,10^ A recent prospective study of 95 consecutive patients undergoing surgery for osteoarthritis of the thumb described a relationship between MP joint hyperextension and thumb column shortening associated with a circular metacarpal head on the lateral view.^25^ The author proposed restoration of the thumb column length in surgery for TM osteoarthritis and the contraindication of trapeziectomy in patients with this type of metacarpal head. These results and the present observations are consistent with reports of improved MP stabilization when TM osteoarthritis is treated by TM prosthesis rather than by trapeziectomy and suspensionplasty because it restores thumb length, corrects presurgical hyperextension deformity, and avoids late postoperative development and the need for revision surgery.^16,24^

Failure to correct MP joint instability may result in the recurrence of first metacarpal adduction, increasing stress on the treated TM joint.^26^ Numerous methods have been proposed to treat MP joint hyperextension deformity, including brachioradialis to EPB tendon transfer,^27^ percutaneous transfixion of the joint,^28^ volar plate advancement with pullout or bone anchor fixation,^29^ sesamoid arthrodesis^30^, capsulodesis,^31^ EPB tenotomy and tendon transfer,^32^ APB transfer to A1 pulley,^33^ percutaneous metacarpal osteotomy with external fixation,^34^ and MP arthrodesis, especially when the deformity exceeds 40°.^35^ However, there is no consensus on the optimal approach. The present data and other published results suggest that hyperextension >40° does not require MP joint arthrodesis except in patients with degenerative joint changes and/or complex instability. Our team previously observed no recurrences in patients who underwent EPB tenodesis^15^ with corrections >50°, and the MP joint retained an acceptable range of motion. Application of this technique in the present model confirms that it corrects hyperextension with respect to stages 3, 4, and 5, reaching extension and MP-ROM values that do not significantly differ from baseline, and that simultaneous treatment with MP flexion tenodesis and trapeziectomy does not modify the MP-ROM.

This study has potential clinical implications for the surgical management of patients with TM osteoarthritis. Preoperative MP joint hyperextension is highly likely to persist or even worsen after trapeziectomy, and this hyperextension can sometimes appear after the intervention. If symptomatic, this deformity is unlikely to be corrected with suspensionplasty or EPB tenotomy and requires an MP joint procedure.

Study limitations include the relatively small number of study specimens and the inability to evaluate in a cadaveric model the effects of neuromuscular control and wound-healing processes on posttrapeziectomy MP joint deformity. In addition, a specific form of suspensionplasty was applied, and outcomes may differ with other techniques. Finally, data were only analyzed in relation to flexion-extension (sagittal plane), and posttrapeziectomy MP joint deformity is three-dimensional and sometimes involves valgus deviation of the first phalanx. The study does not explore how the findings might correlate with clinical outcomes such as pain relief, functional recovery, or long-term joint stability.

## Conclusion

In a cadaveric model of trapeziectomy, secondary thumb column shortening favors an MP joint hyperextension deformity that is not corrected by LRTI or EPB tenotomy and requires an MP joint stabilization procedure.
